# High energy oxidation and organosolv solubilization for high yield isolation of cellulose nanocrystals (CNC) from *Eucalyptus* hardwood

**DOI:** 10.1038/s41598-018-34667-2

**Published:** 2018-11-07

**Authors:** Renli Zhang, Yun Liu

**Affiliations:** Beijing Key Laboratory of Bioprocess, College of Life Science and Technology, Beijing University of Chemical Technology, Beijing, 100029 China

## Abstract

Cellulose nanocrystals (CNC) have been widely used as responsive materials, chiral templates, and tough nano-composites due to its unparalleled properties. Acid and enzyme hydrolyses are extensively employed to prepare CNC. These traditional approaches exhibit inherent limitations of corrosion hazards, time-consuming process, and/or low yield. Herein, irradiation oxidation and organosolv solubilization are conducted to cause rapid degradation with simultaneous crystallization of cellulose to achieve approx. 87% yield of CNC. The morphology, spectroscopic, and stability properties of the as-prepared CNC are characterized through UV-vis spectroscopy, zetal potential, XRD, TEM, DLS, GPC, FT-IR and TGA techniques. The resultant CNC suspension presents unique property with high stability after 9 months storage at 4 °C. Moreover, CNC liquid crystal phase is successfully generated by addition of anions or cations solution to the CNC aqueous dispersion without stirring. The innovative approach in this work opens an avenue to obtain CNC directly from lignocellulosic biomass through irradiation oxidation and organosolv solubilization without acid hydrolysis and washing procedure.

## Introduction

Cellulose, lignin and hemicellulose are the three principal structural components of lignocellulosic biomass. To achieve economic self-sustainability, the coproduction of fermentable sugars and value-added products from biomass has been emerging as the final goal of modern biorefineries^1^. A myriad of excellent articles on this topic has been available in recent years. In 2014, Luterbacher and co-workers^2^ reported laboratory-scale coproduction of soluble carbohydrates and lignin from corn straw, hardwood, and softwood at high yield (70 to 90%) in a solvent mixture of biomass-derived γ-valerolactone (GVL), water, and dilute acid (0.05 weight percent H_2_SO_4_). In 2016, our research group^3^ published investigations wherein GVL/H_2_O solubilization was demonstrated to enhance the fractionation efficiency through high biomass loadings of 30–40 wt.% from agricultural straws, hardwoods and soft woods. In 2017, Yarbrough and co-workers^4^ published exploratory work wherein multifunctional cellulolytic enzymes were used to produce nanocellulose and coproduction of sugars from softwood bleached Kraft pulp. The purposes of those studies were to hydrolyze carbohydrates to achieve high yields of soluble sugars for biofuels production; it was not a favorable outcome if cellulose nanocrystals (CNC) were taken into consideration.

CNC exhibits a great potential in composite materials^5^, electronic and optical devices^6^, and biomedical products^7^. CNC is generally described as shard-like, highly crystalline microfibril fragments with diameters ranging from 3 to 10 nm and lengths from 50 to 500 nm, depending on the source and production method^8^. It was typically obtained by the methods of mineral acids^9^ and enzyme hydrolysis^4^. Reports on enzymatic production of CNC have been fewer in the literatures relative to acid hydrolysis method, which is the dominant strategy for CNC production. The advantages and disadvantages concerning acid and enzyme hydrolysis for CNC production have been described by Li and coworkers^9^. It should be noticeable that, in the case of traditional acid hydrolysis for CNC production, CNC yield was generally fairly low, around 20–50%^9^. The washing process together with the harsh hydrolysis conditions was probably viewed as one of the main contribution to the low yields.

Recently, many new methods have been put forward to produce CNC in the literatures. For instance, Lu and co-workers^10^, Chen and co-workers^11^ presented an efficient approach for extracting CNC from bamboo pulp and fiber using fully recyclable organic acids. Kontturi and co-workers^12^ reported a novel strategy on CNC preparation in hydrogen choride vapor with 97.4% yield from Whatman 1 filter papers. The authors demonstrated that the use of HCl vapor resulted in the rapid degradation and hydrolysis of cotton-based cellulose fibers without practically any mass loss in the cellulose substrate^12^. Lee and co-workers^13^ reported a facile and eco-friendly extraction of cellulosenanocrystals from softwood pulp via electron beam irradiation followed by high-pressure homogenization. However, limited information on CNC isolation directly from lignocellulosic biomass has been available using irradiation oxidation followed by organosolv solublization.

In this present work, using *Eucalyptus* hardwood as model raw feedstock, a facile environment eco-friendly proposal is described to prepare CNC directly from lignocellulose via irradiation oxidation and organosolv solubilization. The organosolv solubilization can result in a rapid lignin-dissolving and high-yield isolation of CNC from cellulose without adding adscititious acid as catalyst. More importantly, an increase in crystallinity occurred, as observed by X-ray diffraction (XRD), with high zeta potential (ZP) value during hydrolysis process. Moreover, the morphology, spectroscopic, and stability properties of the as-obtained CNC are systematically characterized. In the end, CNC liquid crystals phase is successfully produced by adding monovalent (H^+^, Na^+^) and high valency cations (Co^2+^, Zn^2+^, Fe^3+^) into CNC suspension at room temperature without stirring. In comparison with the CNC isolation strategies in the literatures, the merits in our work are: (1) CNC isolation directly from lignocellulosic biomass not from pulp and filter; (2) High yeild (~87%) of CNC obtained without acid hydrolysis and time-consumption washing; (3) High stability of the resultant CNC with no precipitates after 9 months storage; (4) Liquid crystals phases formation with low CNC concentration of 0.5 wt% by adding cation solution.

## Materials and Methods

### Materials

*Eucalyptus* was gifted from Forest Products (Guangxi, China). The contents of cellulose, hemicellulose, and lignin were calculated to be approx. 41%, 15%, and 27% respectively, according to standard NREL Laboratory Analytical Procedures^14^. γ-Valerolactone (GVL, ≥98%), tetrahydrofuran (THF, AR) and dioxane (AR), were purchased from Sigma-Aldrich Co. (St. Louis, MO, USA). Ethanol, NaOH and H_2_SO_4_ were of analytic grade and bought from Beijing Chemical Factory (Beijing, China). Anhydrous pyridine (AR) and phenyl isocyanate (99%) were purchased from Sinopharm Chemical Reagent Co., Ltd (Beijing, China). Sodium hypochlorite (available chlorine ≥8.0%) was from Xilong Chemical Reagent Co., Ltd (Guangdong, China). All chemicals used in this work were used without any further purification.

### CNC isolation from *Eucalyptus* hardwood via organosolv solubilization

Prior to solubilization experiments, *Eucalyptus* wood was irradiated at 800 kGy using ^60^Co-γ as radiation source with the intensity of 9.99 × 10^15^ Bq in the Hunan Irradiation Center (Changsha city, Hunan province, China). The procedures of irradiation pretreatment on *Eucalyptus* were detailed in our previous work^3,15^. The irradiated wood material was milled to  <20 meshes particle size and dried at 105 °C for 4 h. CNC was successfully obtained from irradiated *Eucalyptus* by a three-step process that comprised cellulose fractionation in organosolv mixture solutions, NaClO bleaching and ultrasonic disintegration process. In the first step of fractionation in organosolv mixture solution, residue solid (RS) of cellulose fraction was obtained. In the second step, the separated RS was selected for further bleaching treatment with a mixture solution containing NaClO and NaOH, and the bleached cellulose (BC) was achieved. In the third step, BC samples suspended in distilled water were subjected to ultrasonic disintegration to obtain a uniform dispersion, and finally CNC solution was obtained. During CNC preparation, two types of *Eucalyptus*, irradiated and untreated feedstocks, were compared.

#### Cellulose fractionation from irradiated and untreated *Eucalyptus* in organosolv mixture solutions

40 g of irradiated *Eucalyptus* were loaded into 500 mL high-pressure stainless steel reactor equipped with a stirring impeller. Then, 400 mL of organosolv solutions (including GVL/H_2_O (50:50, vol%), THF/H_2_O (60:40, vol%), dioxane/H_2_O (60:40, vol%)) were subjected to the reactor. Fractionation reaction was started at 170 °C for 1 h by stirring at 400 rpm. After reaction, the reactor was immediately cooled to below 40 °C by flowing cold water through the external heating jacket. The reaction mixture was filtered using 45 *μ*m filter paper to separate the residual solids and the filtrate. The residual solid (RS), assigned as insoluble cellulose fraction, was thoroughly washed with organosolv solution followed with ultrapure water for three times. The RS samples were denoted as I-GRS, I-TRS, and I-DRS corresponding to GVL, THF and dioxane organosolv solutions, respectively.

RS samples from unirradiated *Eucalyptus* were obtained using GVL/H_2_O (70:30, vol.%; 70 mM H_2_SO_4_) and ethanol/H_2_O (65:35, vol%; 100 mM H_2_SO_4_) as organosolv mixture solutions. The other conditions were similar to those from irradiated *Eucalyptus* described as above. The RS samples from unirradiated *Eucalyptus* were expressed as U-GRS and U-ERS related to GVL and ethanol organosolv solutions, respectively.

All as-prepared RS samples were stored at 4 °C for the following CNC preparation.

#### CNC preparation by NaClO bleaching and ultrasonic disintegration process

CNC was successfully obtained from the RS samples by a two-step process that comprised NaClO bleaching and an ultrasonic disintegration process^16^. The conditions of NaClO bleaching were depicted in Table 1. Specifically, the RS samples were selected for further bleaching treatment with a mixture solution containing NaClO and NaOH. The bleaching experiments were performed in a flask at 50 °C for 2 h. The solid/liquid ratio was 1:20 (w/v). After bleaching, the slurry was centrifuged and washed by deionized water until pH neutralization. Then, white-like solid cellulose was obtained and referred as bleached cellulose (BC), which was expressed as I-GBC, I-TBC, I-DBC, U-GBC, and U-EBC, respectively. U-GBC was hydrolyzed with sulfuric acid to extract high crystalline cellulose.

**Table 1:** CNC preparation by NaClO bleaching.

Residual Solids	NaClO (mg/g dried solids)	NaOH	Number of Times	Reaction Conditions
I-GRS	200	1 wt.%	2	50 °C, 2 h, 1:20 (w/v)
I-TRS	200	1 wt.%	3	50 °C, 2 h, 1:20 (w/v)
I-DRS	200	1 wt.%	3	50 °C, 2 h, 1:20 (w/v)
U-ERS	400	2 wt.%	3	50 °C, 2 h, 1:20 (w/v)
U-GRS	400	2 wt.%	2	50 °C, 2 h, 1:20 (w/v)

All obtained BC samples were suspended in distilled water (concentration 0.5 wt.%, calculated by the weight percentage of oven-dried sample to solution) with the total volume of 300 mL. The dispersion was subjected to sonication (600 W, a cylindrical titanium alloy probe (20 mm in diameter), 20 kHz ultrasonication frequency, JY98-IIIDN, Ningbo Scientz Biotechnology Co., Ltd, China) for 80 min to obtain a uniform dispersion, resulting in CNC solution. However, U-GBC and U-EBC dispersions were subjected to sonication for 30 and 100 min respectively to obtain a uniform dispersion. The as-generated CNC suspensions were labeled as I-GCNC, I-TCNC, I-DCNC, and U-ECNC, respectively.

#### Determination of CNC yield

A specified content of the CNC suspension was transferred to a weighing bottle, followed by freeze-dried. Then, the bottle containing the dried CNC powder was weighed. The final CNC yield was obtained through weight difference and calculated as eq. 1.

1 $$CNC\,yield( \% )=\frac{{m}_{1}-{m}_{2}}{{m}_{3}\times C}$$

where, m_1_ is the total mass of dried CNC powder and weighting bottle (mg), m_2_ is the mass of the weighting bottle (mg), m_3_ is the mass of feedstock material (mg), C is the cellulose content of raw feedstock material (%).

### Morphology, spectroscopic, and stability properties of the as-obtained CNC

#### UV-vis spectroscopy observation

The UV-vis spectroscopy of aqueous CNC suspensions (0.5 wt.%) was tested in the wavelength range of 400–900 nm on a UV-vis spectrophotometer (Shanghai YouKe Instrument Co. Ltd, China).

#### X-ray diffraction (XRD) analysis

The XRD patterns were obtained using an X-ray diffractometer (Bruker, Germany) with Ni-filtered Cu Kα radiation at 40 kV and 40 mA. The diffraction data were collected from 2θ = 5−45°, 30 min per sample. The relative crystallinity index (CrI) was estimated using XRD peak height method^17,18^ and calculated as eq. 2.

2 $$Crl( \% )=\frac{{I}_{002}-{I}_{am}}{{I}_{002}}$$

where, I_002_ is the peak intensity of the (002) lattice diffraction at 2θ ≈ 22.3°, which represents both the crystalline and amorphous regions, and I_am_ is the diffraction intensity of amorphous fraction at 2θ ≈ 18.3°.

#### Zeta potential (ZP) determination

All experiments were carried out in dilute CNC suspension (0.5 wt.%). The zeta potential (DLS, Zeta sizer Nano ZS, Malvern instrument) was used to evaluate the colloidal stability (zeta potential and size) of CNC suspensions based on electrophoretic light scatting at 25 °C.

#### Dynamic light scattering (DLS) measurement

CNC samples were dispersed in distilled water to achieve 0.5 wt.% suspensions. Size distribution experiments of CNC were performed in duplicate with a dynamic light scattering evaluator (DLS, Zeta sizer Nano ZS, Malvern instrument) at 25 °C.

#### Gel Permeation Chromatography (GPC) analysis

Prior to GPC analysis, cellulose samples were derivatized using anhydrous pyridine and phenyl isocyanate according to the procedures reported by Meng *et al*.^19^. The derived cellulose samples were dissolved in THF solvent (Baker, HPLC grade) with the final concentration of 10 g/L. The mixture solutions were filtered with 0.22 *μ*m nylon membrane syringe filters. GPC analysis was carried out using an 1515 GPC instrument (Waters, USA) with three columns (Polymer Laboratories, 300 × 7.5 mm) packed with polystyrene-divinylbenzene copolymer gel (10 *μ*m beads) with nominal pore diameters of 10^4^, 10^3^, and 50 Å. THF was used as eluent with the flow rate of 1.0 mL/min. An injection volume was 50 *μ*L. A refractive index (RI) detector was used for measurement. Retention time was converted into molecular weight (Mw) by applying a calibration curve using polystyrene standards (molecular weight is ranging from 500 to 4 × 10^6^ Da). Degree of polymerization (DP) of cellulose was calculated using the molecular weight dividing by 519, the molecular weight of cellulose tri-carbanilate monomer^20^.

#### Fourier Transform infrared spectroscopy (FT-IR) Analysis

FT-IR spectra were recorded using a FT-IR spectroscopy (VERTEX 80 v, Bruker, USA). The spectra were recorded from 600 to 2000 cm^−1^ at a resolution of 1 cm^−1^.

#### X-ray photoelectron spectroscopy (XPS) analysis

The XPS analyses were conducted on an ESCALAB 250 XPS (Thermo Fisher Scientific, USA). The data were acquired using twin anode Al Kα (300 W), pass energy of 100 eV for survey, 30 eV for high resolution scans. The analyzed areas were 500 ìm × 500 ìm. The carbon element signals were deconvoluted (within 0.2 eV) into C1 (285.00 eV), C2 (286.55 eV), C3 (287.88 eV) and C4 (288.81 eV) signals using PEAK Fit (Version 4.1).

#### Transmission electron microscopy (TEM) images

TEM images of the resultant CNC were observed on a HT7700 Hitachi TEM (Hitachi, Japan). Sample was diluted to 0.01 wt.% and drop-cast onto the freshly glow-discharged carbon-coated copper grids. The sample was negatively stained with 2 wt.% aqueous uranyl acetate for 5 min. After removal of the excess solution, the sample was dried at room temperature prior to TEM imaging. The sizes of CNC were measured using an image analysis system (Image J version: 2.0.0-rc-43/1.50e).

#### Thermal gravimetric analysis (TGA) curves

The thermal stability of the cellulose samples was characterized with a DTG-60A thermal gravimetric analyzer (Shimadzu, Japan). The samples were heated from 40 to 1000 °C at a heating rate of 10 °C·min^−1^ in nitrogen atmosphere with the flow rate of 30 mL·min^−1^.

#### Storage stability of CNC suspension

0.5 wt.% of CNC suspension was stored at 4 °C for 9 months to evaluate its stability through UV-vis observation, DLS and ZP measurements. The as-prepared CNC suspension was expressed as the fresh CNC suspension, while the CNC suspension stored for 9 months was expressed as the mature CNC suspension.

### Fabrication of CNC liquid crystal phase

CNC liquid crystal phase was fabricated by addition of monovalent (H^+^, Na^+^) and high valency cations (Co^2+^, Zn^2+^, Fe^3+^) to the CNC aqueous dispersion as described by Zander *et al*.^21^. Briefly, 0.5 wt.% of CNC dispersion (200 *μ*L) was put into a 2 mL centrifugal tube, and then 100 *μ*L of 0.1–1.0 M aqueous solution of ion (acetic acid, HCl, NaCl, NaOH, Co(NO_3_)_2_, Zn(SO_4_)_2_, FeCl_3_) was dropwise added into the tube at room temperature without stirring. After a few minutes, the solutions on the top were decanted and liquid crystal phase could be rapidly observed in the inverted vials. Polarized optical microscopy (POM) images of CNC liquid crystal phase were performed on a Motic BA300-POL microscope (Motic China Group Co., Ltd., Xiamen, China).

## Results and Discussion

### Effect of organosolv solubilization on cellulose fractionation from biomass

**Figure 1:**
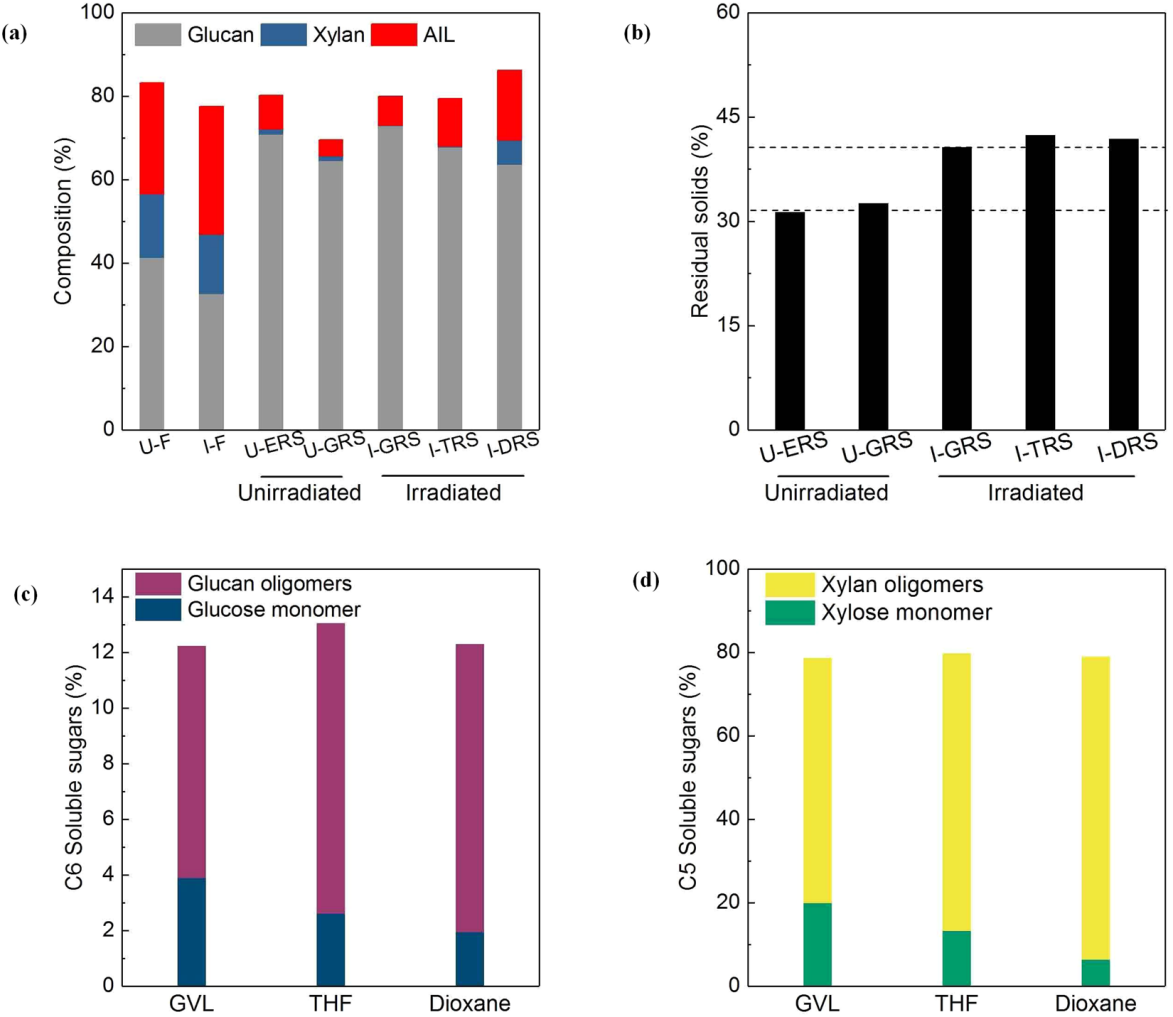
The effect of solubilization systems on chemical composition of the lignicollulose and soluble sugars in eluent. (**a**) The effect of irradiation pretreatment with or without organosolv solubilization; (**b**) The yield of residue solid after organosolv solubilization; (**c**) C6 concentration in organosolv system; (**d**) C5 concentration in organosolv system. U-F means untreated feedstock; I-F expresses irradiated feedstock.

The goals of organosolv solubilization are to degrade and crystallize of cellulose from *Eucalyptus* through delignification and removal of hemicellulose. The effects of different organosolv systems, GVL/H_2_O (50:50, vol.%), THF/H_2_O (60:40, vol%), dioxane/H_2_O (60:40, vol%), on RS cellulose yields were evaluated through biomass component variance. The experiments were conducted in duplicate and the average data are depicted in Fig. 1.

As seen from Fig. 1a, approx. 50–90% of the total lignin and 80–100% of the hemicellulose were removed from the *Eucalyptus* via organosolvents solubilization (Fig. 1a). It indicates that the selected three organosolv systems are suitable for solubilization solutions. As expected, the efficiency of delignification and hemicellulose removal seems to vary with organosolvent systems. In comparison with THF and dioxane systems, GVL/H_2_O solubization achieves about 91% of delignification and 100% of hemicellulose removals from *Eucalyptus*. It is thus speculated that the capacity of solubilization effect is dependent on the used solvents^3^.

In case of unirradiated feedstock, approx. 30% of residual solids (RS) are achieved. For irradiated material, the yield of RS is approx. 40% (Fig. 1b). This phenomenon is attributed to the fact that the irradiation pretreatment generates reactive oxygen free radicals (O_2_^−^), which was detected by EPR (Fig. S1). These radicals are responsible for the oxidization of hydroxy groups on the aromatic rings of lignin and parts of hemicellulose to carboxyl groups. The main feature peak of carbonyl groups (C=O) toward irradiated samples at 1730 cm^−1^ was observed whilst it does not appear in the original sample (Fig. S2). This result is good concision with the XPS pattern of irradiated sample (Fig. S3). The amount of carboxylic groups allegedly by irradiation was quantified by conductometric titration^22^, and the value is 0.4 mmol/g CNC. The extent of irradiation-mediated oxidation is almost equivalent to the TEMPO-mediated^22,23^, electron beam-mediated^11^, and oxalic acid-mediated oxdition^13^. The irradiation oxidation was suitable for isolation of cellulose because the oxidation agent is more aggressive towards lignin and hemicellulose, whereas cellulose is hardly decomposed under the mild conditions^16^.

After irradiation pretreatment, the irradiation-mediated carboxyl groups can act as active acid moieties to hydrolyze both amorphous regions of cellulose fraction to C6 (9.6%, 180 mg/L) and hemicellulose to C5 sugars (82.3%, 600 m g/L) in orgnosolv systems. These sugars were detected by HPLC and the data are show in Fig. 1c,d. In our initial works, irradiation pretreatment would decompose the lignocellulosic stubborn structure, resulting in enhancement of the downstream enzymatic hydrolysis, yeast fermentation and ethanol conversion of cellulose^3,15,24–26^. In 2016, Zhou and co-workers reported a novel strategy of lignocellulose fractionation at solid biomass loadings up to 40 wt.% through irradiation assisted self-catalysis in GVL/H_2_O (40:60, vol.%) without adding mineral acids^3^. These articles showed that irradiation pretreatment had benefit effect toward delignification and hemicellulose removal.

### Effect of organosolv solubilization on the characteristics of CNC

**Figure 2:**
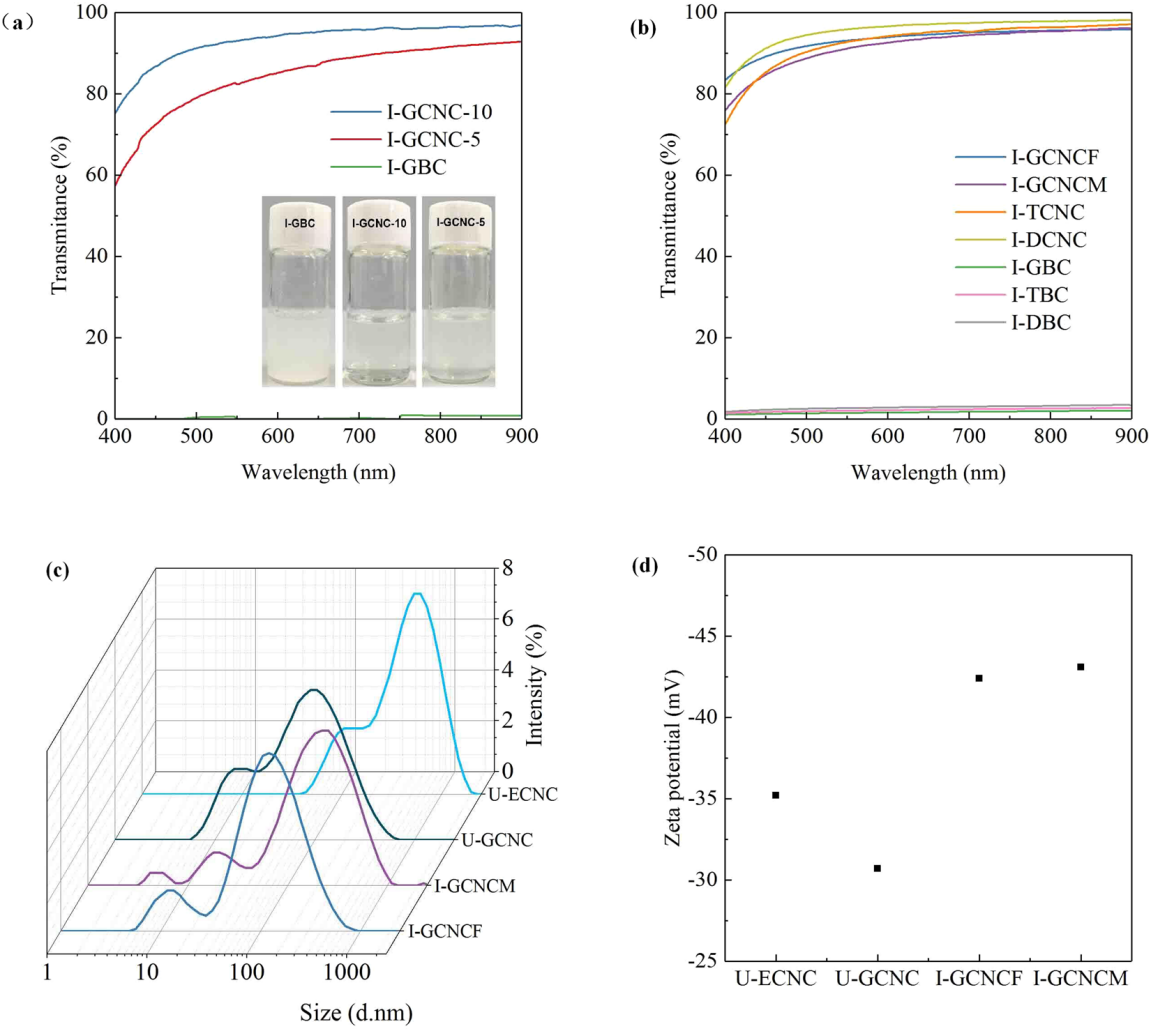
(**a**) UV-vis transmittance spectra of 0.5 wt.% CNC suspensions;(**b**) UV-vis transmittance spectra of 0.5 wt.% CNC suspensions obtained from different co-solvent systems; (**c**) DLS measurement of the CNC suspensions; (**d**) Zeta potential plot of the CNC suspensions. I-GCNCF means the fresh CNC preparation; I-GCNCM refers to CNC suspension for 9 months storage.

The as-prepared bulk cellulose was subjected to NaClO bleaching and an ultrasonic disintegration process to achieve CNC. Using I-GCNC as an example, Fig. 2a shows that a substantial change is observed in the dispersion of the CNC in water before and after ultrasonic disintegration (30 mL suspension was sonicated at 600 W for 5 and/or 10 min). The transmittance of CNC suspension is dependent upon sonication time. UV-vis observation indicates that 0.5 wt.% CNC suspension is reaching 95% of transmittance at 900 nm regardless of organosolvent (Fig. 2b). After 9 months storage at 4 °C, the CNC suspension is still transparent via UV-Vis observation and no precipitates are observed. DLS analysis reveals that the sizes of the CNC are 100–250 nm for 9 months storage and is small enough to be highly dispersed in aqueous solutions (Fig. 2c). The surface charges of the fresh CNC (I-GCNCF) and the mature CNC (I-GCNCM) for 9 months storage were detected by ZP measurements, which both show negative ZP values of about -43 mV (Fig. 2d). Obviously, CNC prepared from unirradiated *Eucalyptus* has lower ZP value (−30~−35 mV) than that from irradiated raw material. Low ZP value suggests weak electrostatic repulsive forces among the CNC fibers, which are easily aggregated together to form large precipitates. This hypothesis is confirmed through the UV-vis observation of CNC suspensions from unirradiated *Eucalyptus* (Fig. S4). On the contrary, high ZP values (−43 mV) of CNC suspension isolated from irradiated *Eucalyptus* suggest strong electrostatic repulsive forces among the CNC fibers, hampering to aggregate together of CNC fibers. The reasonable explanation is the fact that hydrophilic carbonyl groups or enol ethers are formed on cellulose structure after irradiation oxidation toward cellulose, these carbonyl groups (C=O) was detected by FTIR and XPS (Figs S2 and S3).

**Figure 3:**
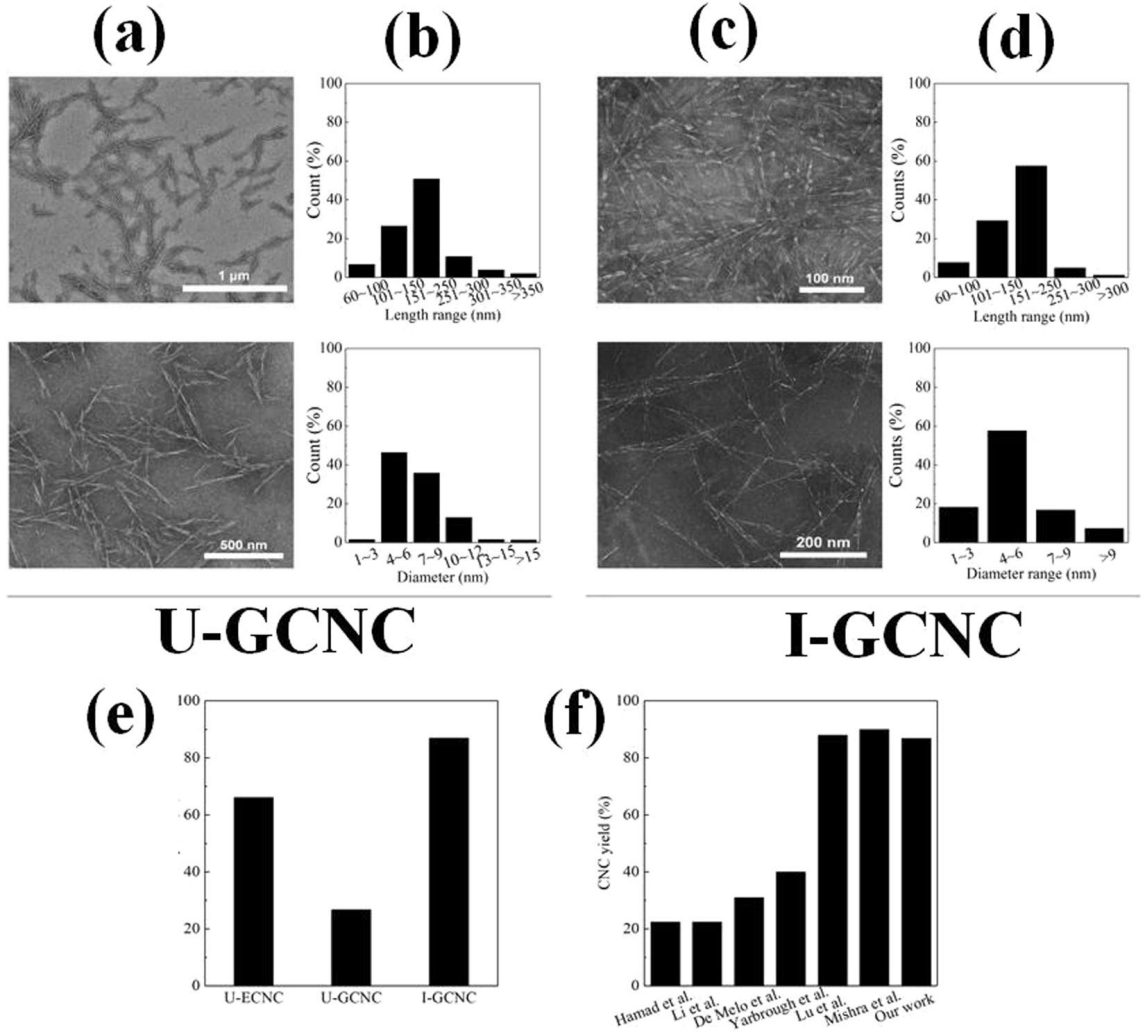
(**a**) TEM images of the CNC morphologies of U-GCNC; (**b**) size distribution of U-GCNC; (**c**) TEM images of the CNC morphologies of I-GCNC; (**d**) size distribution of I-GCNC; (**e**) CNC yield by different treatment; (**f**) Comparison of CNC yield in our work with that reported in the literature.

Morphology and nano-size observation of the obtained CNC in this work were conducted by TEM. As seen from Fig. 3a–d, the widths of CNC range 4 ~6 nm and its lengths are 150~250 nm. However, if the *Eucalyptus* was treated with irradiation, the widths and lengths of the resultant CNC (I-GCNC) were slight narrower and shorter than the unirradiated samples (U-GCNC). In comparison with the references, the width of CNC from *Eucalyptus* hardwood in our work is one fifth smaller than that measured by Elazzouzi-Hafraoui *et al*.^27^ (20~30 nm) and Lee *et al*.^13^ (~26 nm), and similar width with that measured by Peyre *et al*.^22^ (7 nm), and three times larger than that measured by Abushammala *et al*.^28^ (1.9 nm). These differences are explained by supposing that these authors have measured the width of elementary crystalline which is not probably correspond to single crystals, but one particle inherently constituted of several crystallites.

As control, acid hydrolysis method was employed to prepare CNC from filter paper and irradiated *Eucalyptus*^9^. TEM images show that the widths and lengths of CNC obtained by sulfuric acid hydrolysis filter paper are similar to those CNC prepared by organosolvents solubilization (Figs S5 and S6). It is worthily noticed that acid approach for CNC preparation exhibits inherent limitations of corrosion hazards and environmental issues. Also, acid hydrolysis for CNC preparation is a time-consuming process due to long time dialysis (about 4 days). As seen from Fig. 3e,f, the yield of CNC obtained by irradiated feedstock by organosolv solubilization is calculated to be approx. 87%, it is much higher than those obtained by concentrated sulfuric acid hydrolysis and peroxide solvothermal methods^9,16^. and almost equivalent to those of CNC obtained by TEMPO oxidation^29^ and by simultaneous mechanochemical activation and phosphotungstic acid hydrolysis^10^. Some researchers reported much higher CNC yield (97.4%) could be isolated from Whatman 1 filter papers in hydrogen chloride vapor^12^.

**Figure 4:**
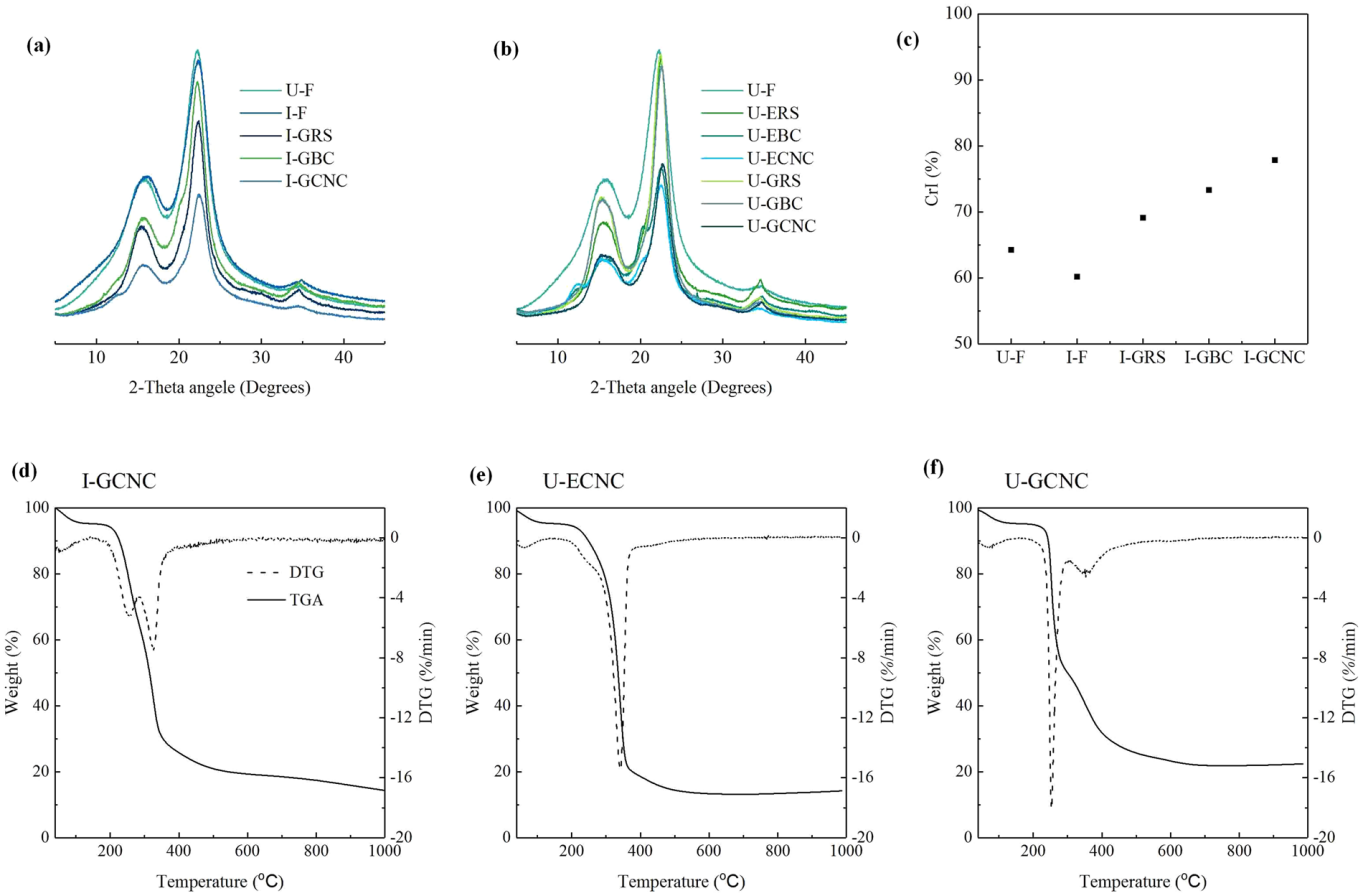
(**a**) XRD patterns; (**b**) crystallinity of the CNC from irradiated material; (**c**)TGA curve of I-GCNC; (**d**) DTG curve of I-GCNC.

The crystallinity and thermal stability of the obtained CNC were analyzed using XRD and TGA methods. As seen in Fig. 4a–c, all XRD patterns exhibit the typical diffraction peaks around 2θ ≈ 18.3° and 22.3°, which confirm that the crystal lattice type I of native cellulose is preserved although raw material is treated through a series of irradiation oxidation and organosolv solubilization. The CrI, as a key factor in influencing the mechanical and thermal properties, was estimated according to the peak height method method^30^. The CrI of the irradiation oxidized samples (I-F) is estimated to be 60.1%, showing 4.1% decrease in comparison with the raw feedstock (U-F, 64.2%). The decrease in the CrI value undoubtedly is caused by the oxidation depolymerization of cellulose, indicating that irradiation oxidation has a destructive effect on the amorphous region and imperfect crystalline region of cellulose. This phenomenon is confirmed through molecular mass weight (Mw) variance after a series of treatments (Table S1). As seen in Table S1, DP_w_ values of cellulose are decreasing from 250 for native cellulose to 52 for irradiated sample. After GVL/H_2_O solubilization, the CrI (69.1%) of CNC (I-GRS) is slight higher than native cellulose (64.2%), indicating that the removal of lignin and hemicellulose are occurred due to solvent solubilization effects. The CrI value of bleached cellulose (I-GBC) increases to 73.3% after NaClO treatment because of further delignification. After ultrasonication, the type I crystal lattice and CrI of the obtained CNC is unchanged (Fig. 4a), but the CrI value (77.8%) only slightly increases by 6.5% in comparison with that of I-GBC sample (73.3%). Fig. S7 show that the tendency of Crl values of the CNC obtained from unirradiated *Eucalyptus* is similar to those from irradiated feedstock.

The TGA curves of the resultant CNC are shown in Fig. 4d–f. Cellulose thermal degradation procedure involves dehydration, depolymerization and decomposition of glycosyl units^10^. In other words, the TGA curve of lignocellulosic biomass has three characteristic regions: volatiles removal was attribute to mass loss, the removal of oxidation and other volatile materials was belonged to the second mass loss, and the third mass loss was attributed to the oxidation of residual coal occurs^31^. In all cases (I-GCNC, U-ECNC, U-GCNC), a small weight loss is found in the range of 25–120 °C due to the humidity evaporation or low molecular weight compounds remaining from the isolation procedures. In cases of all samples, the starting decomposition temperature occurs approx. 220 °C due to the decomposition of residual pectins and hemicellulose. At ca. 350 °C, this is associated with largest mass loss due to cellulose decomposition. At 400 °C almost all cellulose is pyrolyzed, and the solid residuals at 1000 °C are close to 18–25 wt.%. TGA thermograms show evidence of residual non-cellulosic matter^32^. As a result, either irradiation oxidation treatment or NaClO bleaching treatment shows a negative impact on the crystalline region of cellulose. This phenomenon is in good accordance with that reported by Li and co-workers^16^.

### CNC liquid crystals phase formation and optical properties

**Figure 5:**
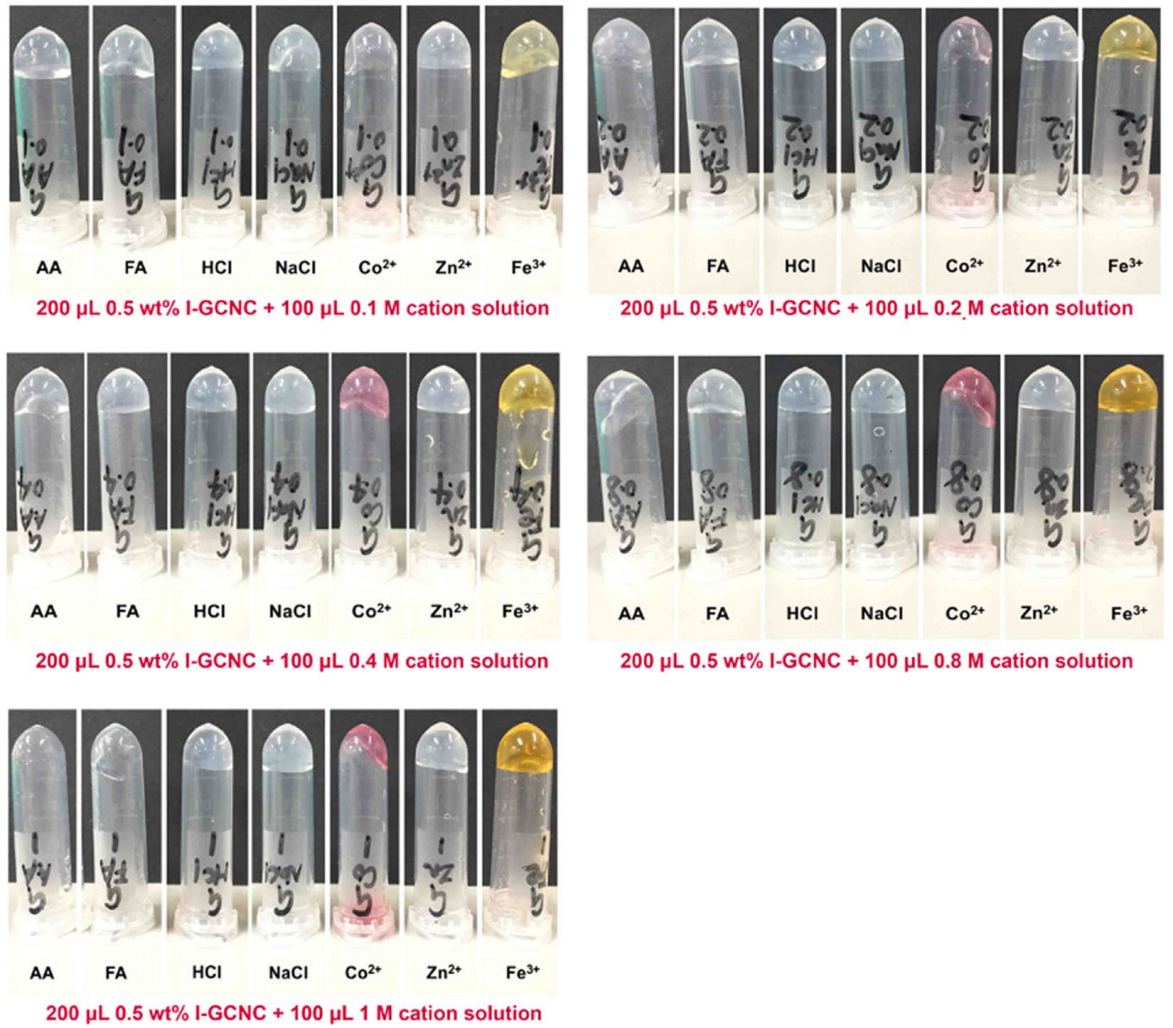
Photos of the CNC hydrogels in an inverted vials by adding 100 *μ*L of cations solution with concentrations ranging from 0.1 M to 1.0 M to the 0.5 wt.% CNC dispersion.

**Figure 6:**
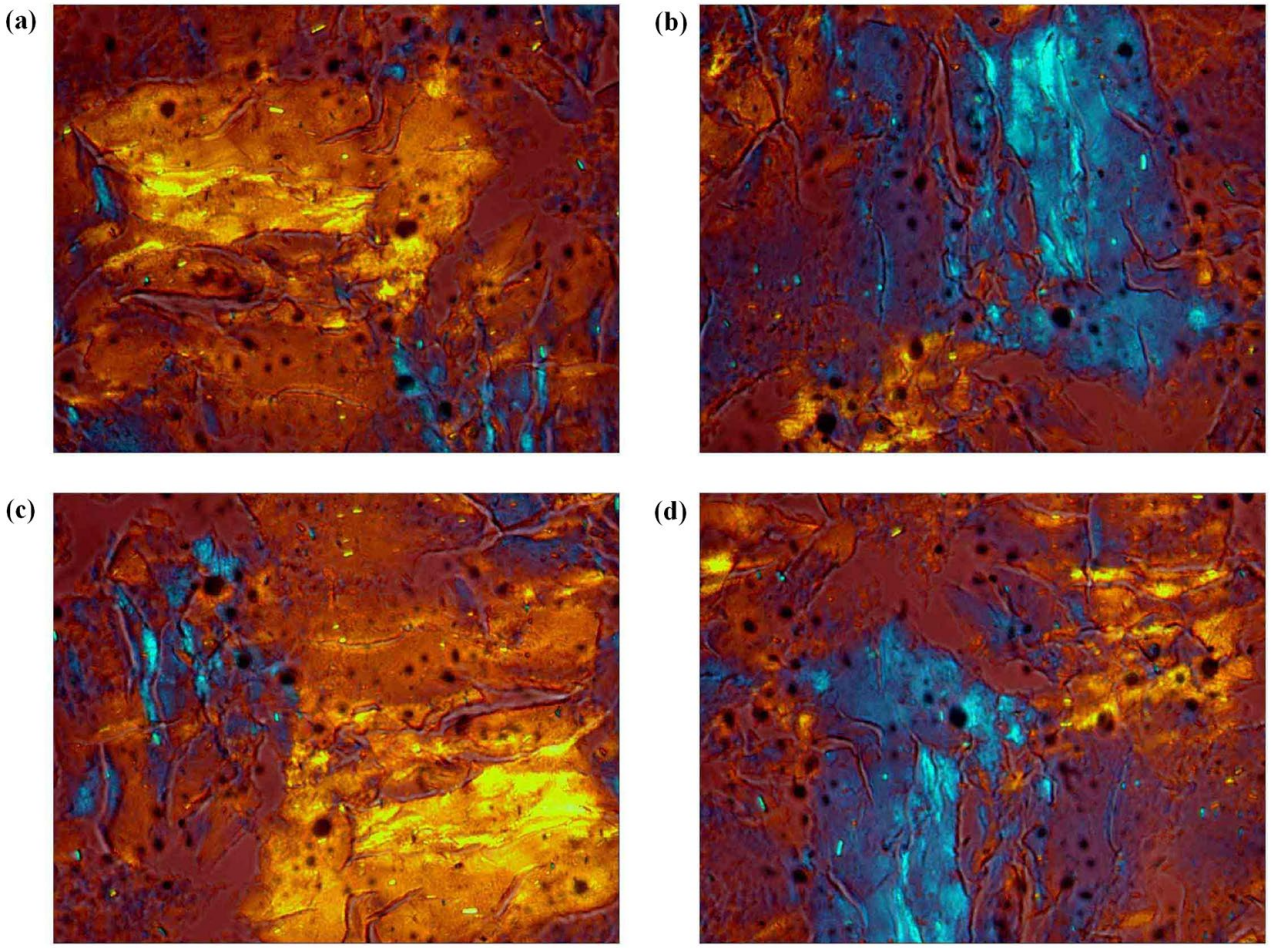
(**a**) POM image of liquid crystal phase generated by adding 100 *μ*L of 0.1 M acetic acid to 0.5 wt.% CNC dispersion; (**b**) POM image of liquid crystal phase for 90° clockwise rotation; (**c**) POM image of liquid crystal phase for 180° clockwise rotation; (**d**) POM image of liquid crystal phase for 270° clockwise rotation.

CNC liquid crystals phase was produced by diffusing monovalent (H^+^, Na^+^) and high valency cations (Co^2+^, Zn^2+^, Fe^3+^) into the CNC dispersion, through binding affinity of cations with carboxylate groups of CNC, the CNC gelation process was initiated^33^. As seen from Fig. 5, rapid gelation of CNC is triggered by adding 100 *μ*L of cations solution to the 0.5 wt.% CNC dispersion, which was observed by inverting the vials with the gels. The concentration of cation solution is ranging from 0.1 M to 1.0 M. Unlike the preceding CNC aqueous dispersion, the CNC liquid crystals phase had no fluidity and maintained shape even with slight shaking. Dong and co-workers demonstrated that the driving force of gelation was proposed to be a screening effect of the repulsive potentials on the fibril surfaces by strong cation ion-carboxylate bonding^34^. As observed in Fig. 6a–d, polarized optical microscopy (POM) images show that CNC liquid crystal phase exhibits optical activity with a shift in color from gold to blue through clockwise rotation of 90° for liquid crystal phase. It is attributed to CNC ordered arrangement^35^. In comparison with the references, low CNC concentration (0.5 wt.%) in our work can form liquid crystals phase^36,37^. High polydispersity of CNC rods promote the formation of liquid crystal phase and arrest gel-like glassy states^38^. The strength of the gel depends on the concentration and type of cation ions. From Fig. 5, when 1 M of acetic acid or formic acid are added into the CNC suspension, the strength of CNC gel is weak, however, 1 M of HCl, Na^+^, CO^2+^, Zn^2+^ and/or Fe^3+^ are added into the CNC suspension, the CNC gel is much stronger due to the strong repulsive ion strength for gel formation^38^. Therefore, CNC is an ideal material as the chiral nematic liquid crystal template for optical devices, sensors and chiral separation^39^.

## Conclusions

This study reports a novel strategy of organosolv solubilization to achieve CNC with the yield of approx. 87% through rapid degradation with simultaneous crystallization of cellulose. The resultant cellulose is 4–6 nm wide and 150–250 nm long, with high polydispersity of 25–63. CNC properties are comprehensively characterized by TEM, DLS, ZP, UV, XRD, XPS, and FT-IR. More importantly, no mineral acids are used through the whole CNC preparation process. Therefore, the approach proposed in our work is a sustainable and environment eco-friendly alternative for CNC preparation. The CNC suspension exhibits high stability, and behaves as a chiral nematic lyotropic liquid crystal with a shift in color. Future work will be done to generate the CNC gels with highly tunable optical properties, mechanical properties, and chemical selectivity.

## Electronic supplementary material

Supplementary Information
