# Supplementary material for: High energy oxidation and organosolv solubilization for high yield isolation of cellulose nanocrystals (CNC) from Eucalyptus hardwood
Source: Sci Rep. 2018 Nov 7;8:16505. doi: 10.1038/s41598-018-34667-2 (PMC6220251; doi:10.1038/s41598-018-34667-2)
Supplement: Supplementary file 1 — Supplementary Information [file 41598_2018_34667_MOESM1_ESM.docx]

**SUPPLEMENTARY INFORMATION**

**High energy oxidation and organosolv solubilization for high yield isolation of cellulose nanocrystals (CNC) from *Eucalyptus* hardwood**

Renli Zhang, Yun Liu*

**Figures LEGENDS**

**Fig. S1** Activation oxygen free radicals monitored by EPR spectra of *eucalyptus* irradiated at 600 kGy.

**Fig. S2** UV-vis transmittance spectra of 0.5 wt% CNC suspensions from untreated *Eucalyptus* material.

**Fig. S3** FTIR curves of irradiated and untreated feedstocks (a), the bleached celluloses (b) and CNC extracted from irradiated and untreated feedstocks through organosolv solubilization (c). Wavelength at 1739 cm^-1^ is the strech vabrication of -OH groups.

**Fig. S4** XPS curves of CNC extracted from the irradiated feedstock.

**Fig. S5** TEM images show the morphology and size distribution of the CNC from filter paper obtained by sulfate acid hydrolysis method.

**Fig. S6** The TEM images of CNC from untreated *eucalyptus* obtained by ethanol/H_2_O (65:35, vol%) solubilization with adding 70 mM H_2_SO_4_ as catalyst.

**Fig. S7** The crystallinity index value calculated from XRD patterns for the CNC obtained from untreated *eucalyptus*

**Fig. S1**


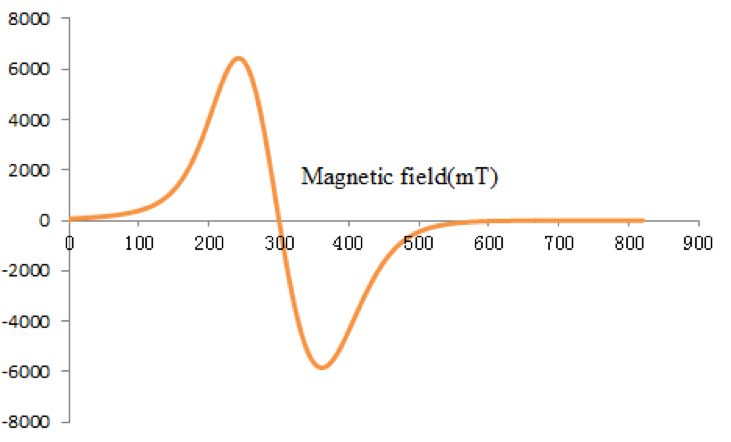


**Fig. S2**


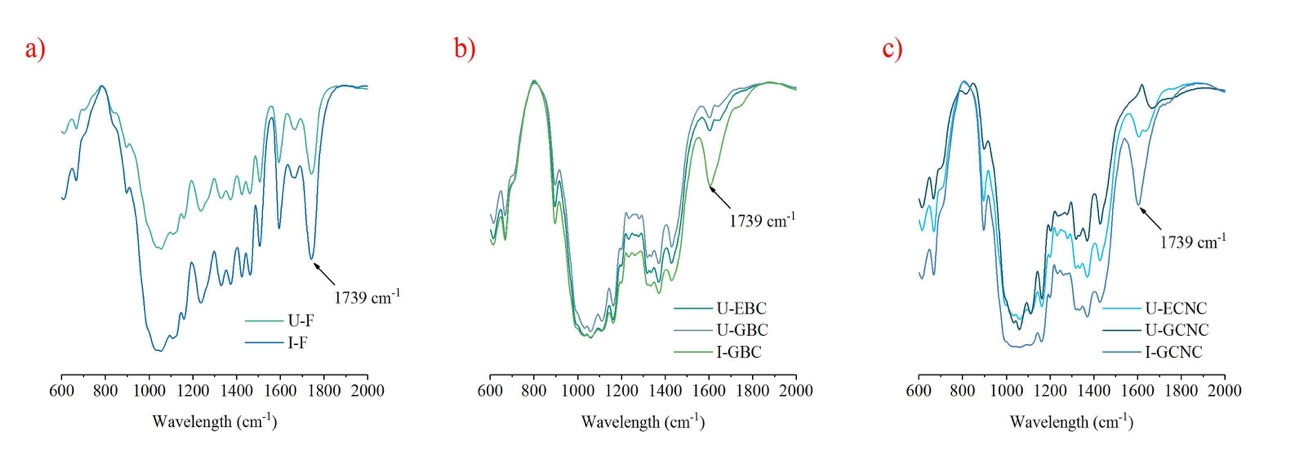


**Fig. S3**


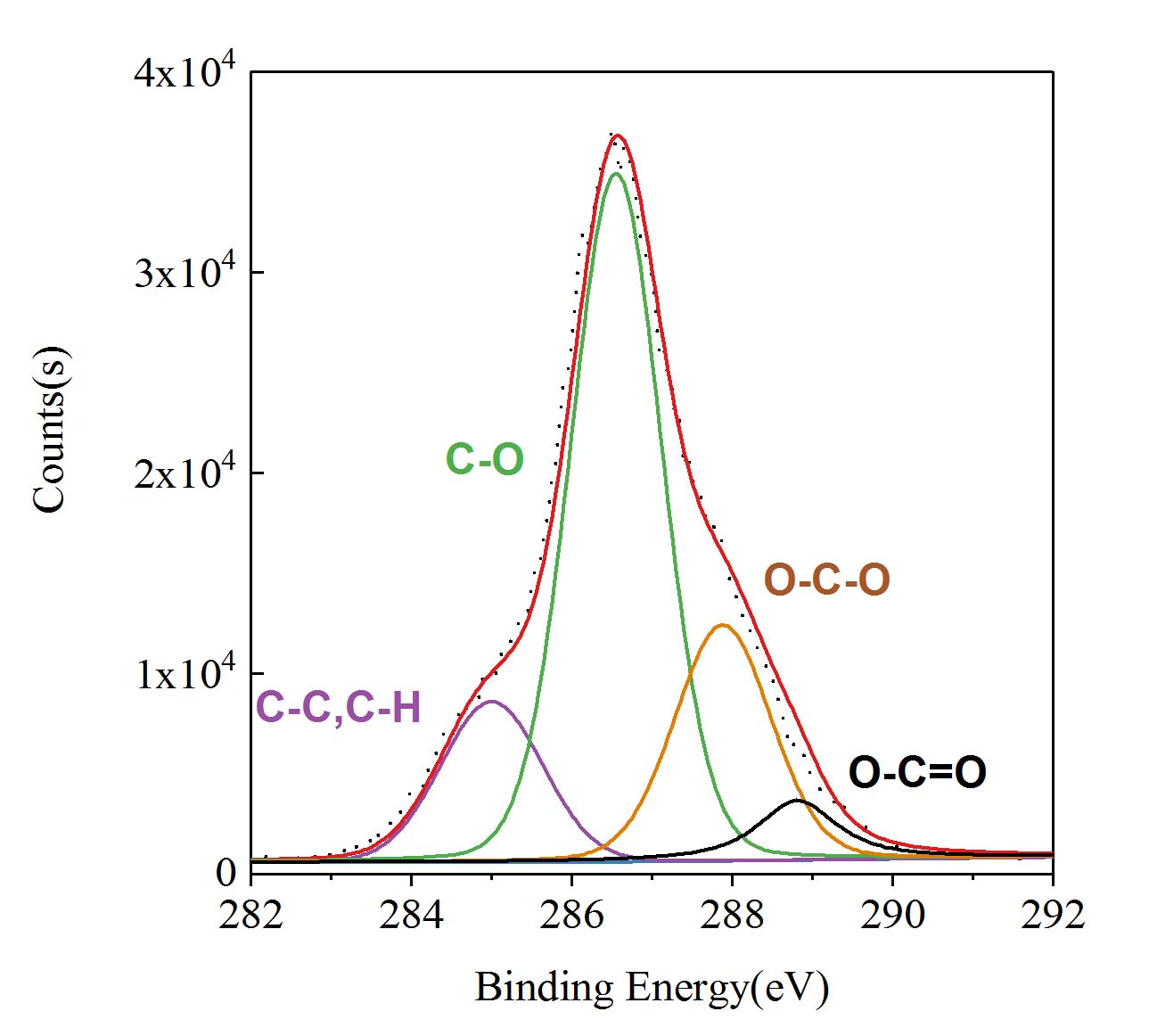


**Fig. S4**


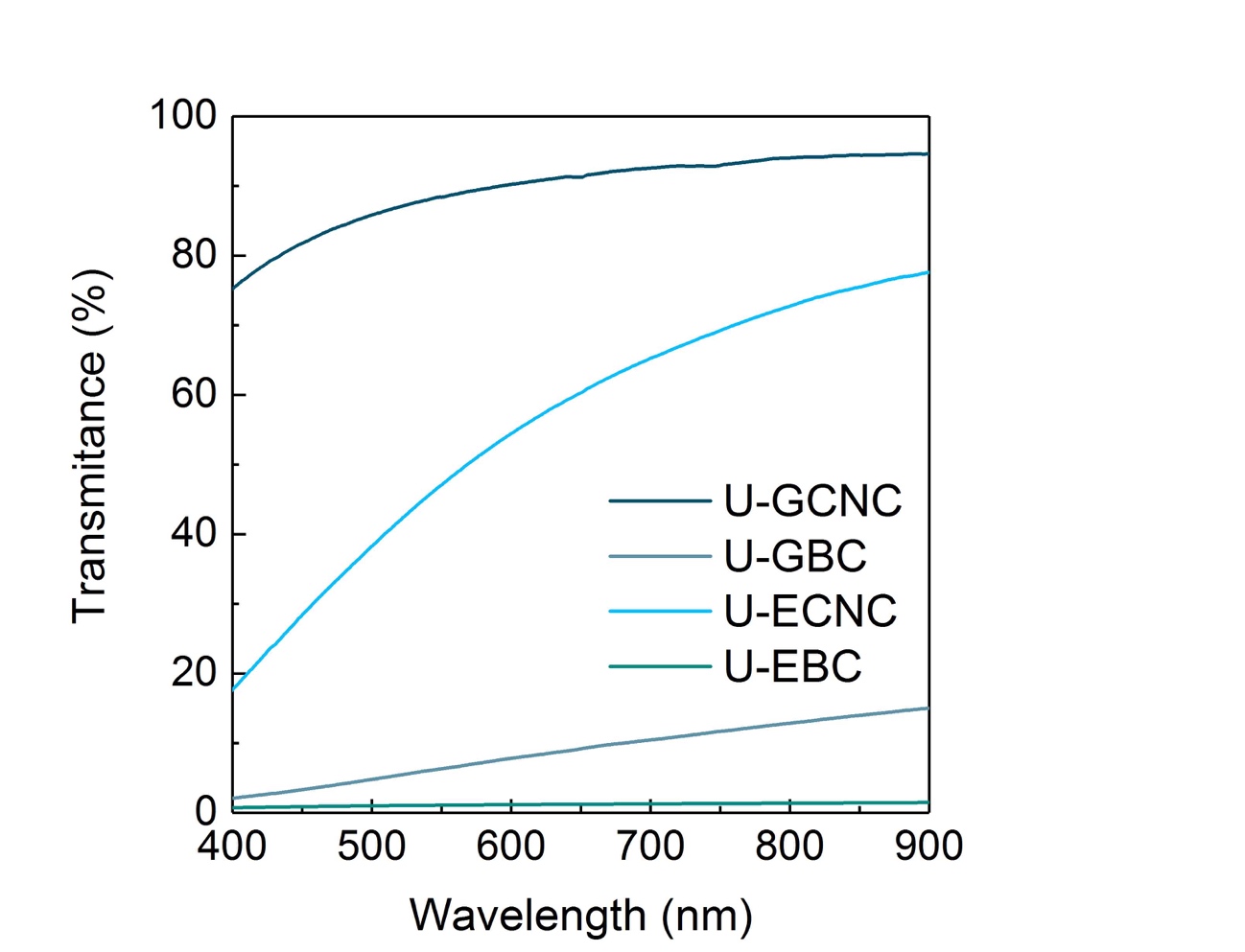


**Fig. S5**


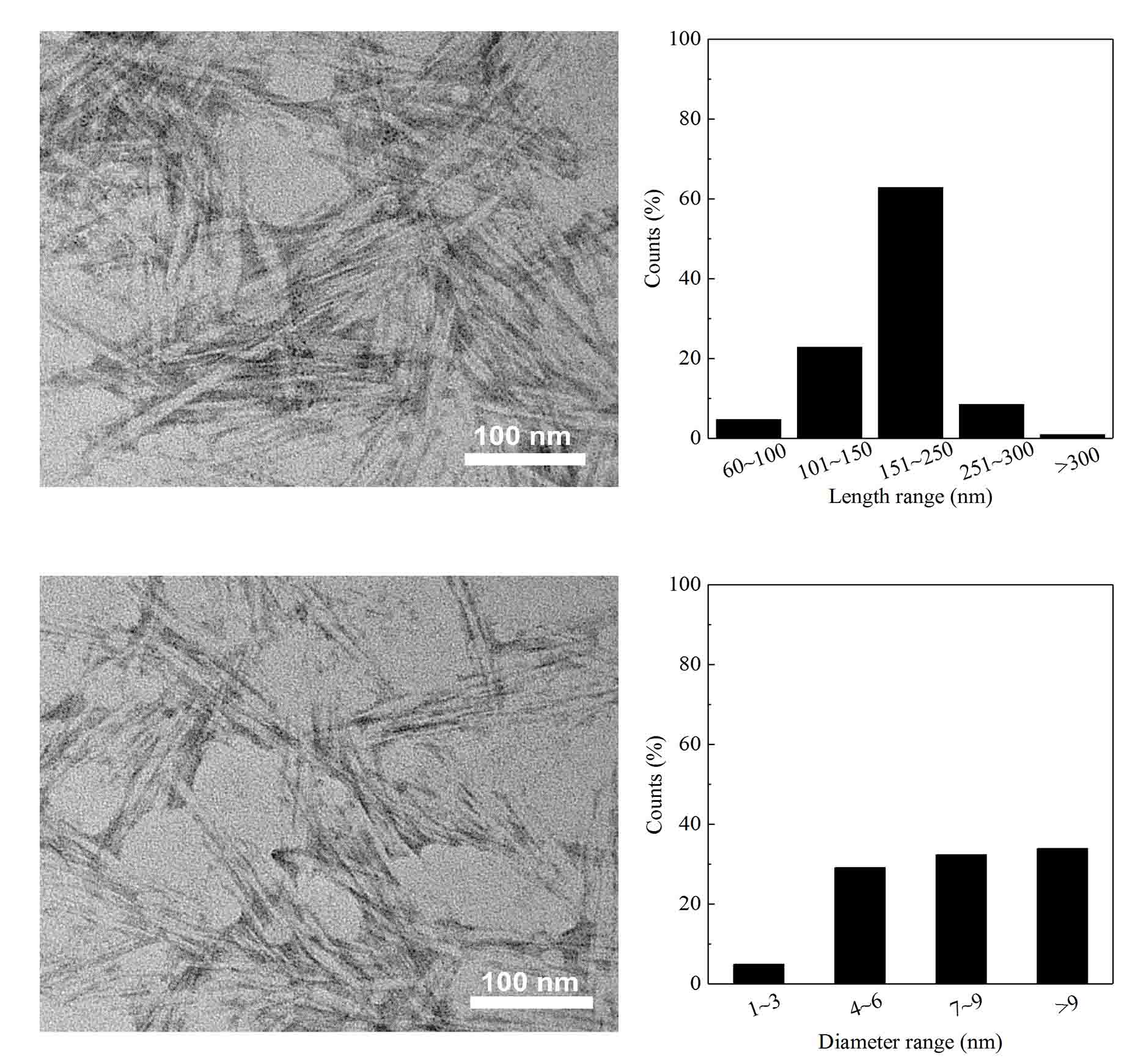


**Fig. S6**


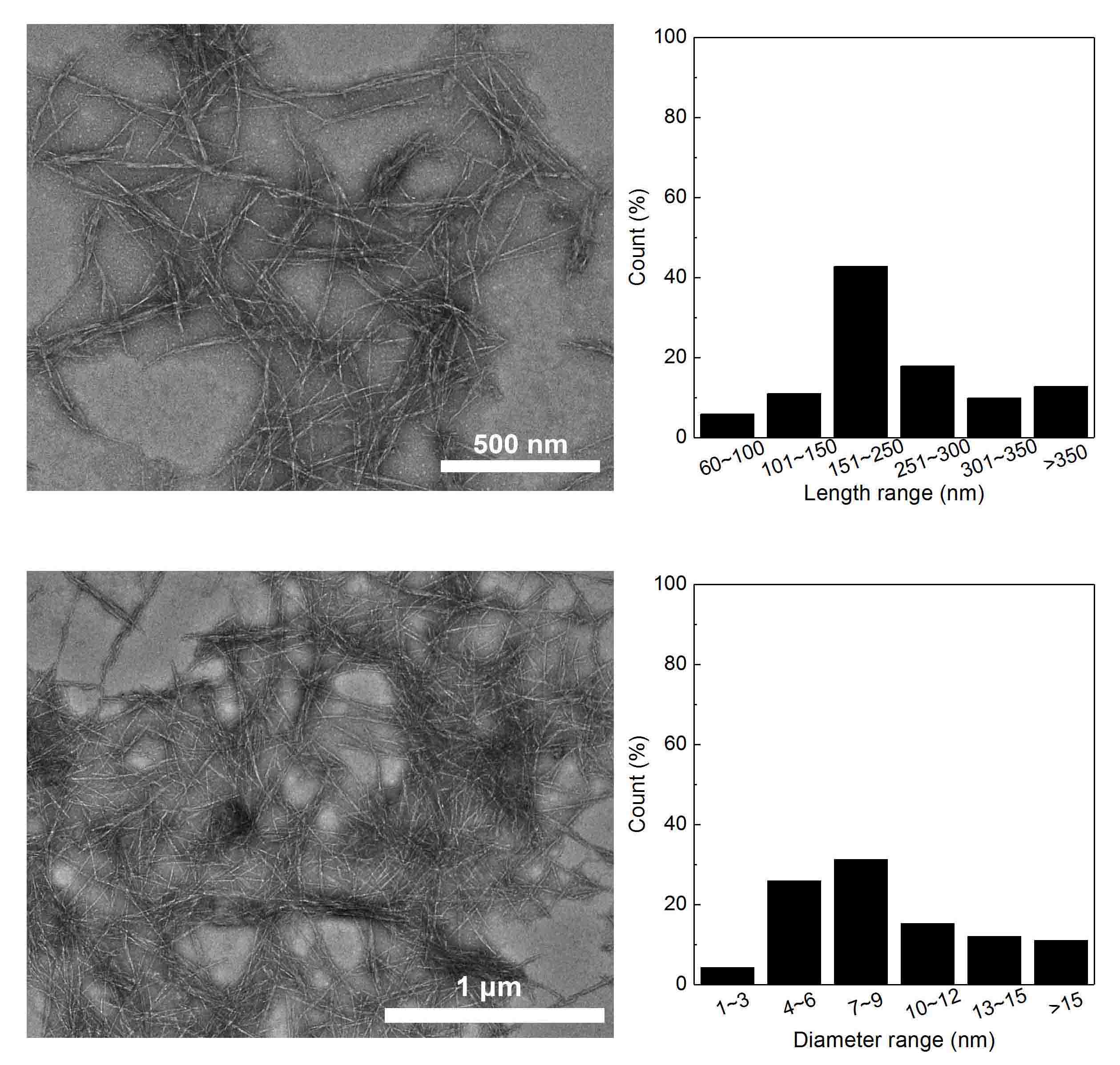


**Fig. S7**


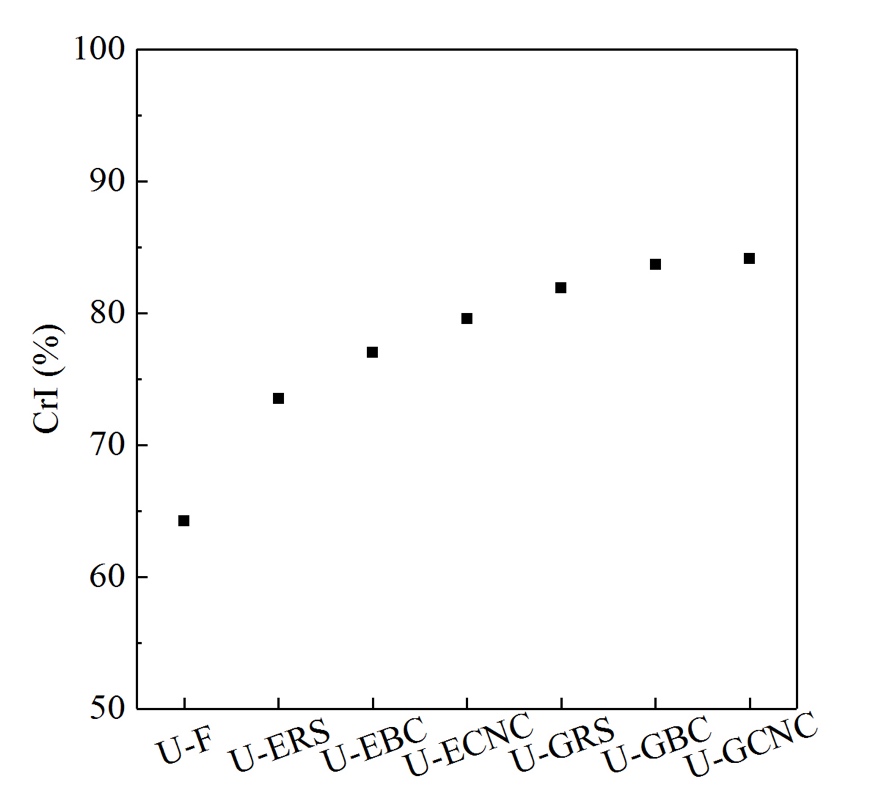


**Table S1** Molecular mass weight and degree of polymerization of all types of bleached cellulose (BC) and cellulose nanocrystalline (CNC)

| Samples | M_n_(g/mol) | M_w_(g/mol) | PI | DP_w_ |
| --- | --- | --- | --- | --- |
| U-EBC | 41436 | 96527 | 2.3296 | 186 |
| U-ECNC | 41073 | 93304 | 2.2716 | 180 |
| U-GBC | 49388 | 129639 | 2.6249 | 250 |
| U-GCNC | 29500 | 52523 | 1.7805 | 101 |
| I-GBC | 11140 | 26705 | 2.3971 | 52 |
| I-GCNC | 10836 | 26895 | 2.4820 | 52 |

DP_w_=M_w_/519 PI=M_w_/M_n_
